# Review of Current Methodological Approaches for Characterizing MicroRNAs in Plants

**DOI:** 10.1155/2009/262463

**Published:** 2009-10-08

**Authors:** Turgay Unver, Deana M. Namuth-Covert, Hikmet Budak

**Affiliations:** ^1^Biological Sciences & Bioengineering Program, Faculty of Engineering and Natural Sciences, Sabanci University, Orhanli, Tuzla, 34956 Istanbul, Turkey; ^2^Department of Agronomy and Horticulture, University of Nebraska-Lincoln, Lincoln, NE, USA

## Abstract

Advances in molecular biology have led to some surprising discoveries. One of these includes the complexities of RNA and its role in gene expression. One particular class of RNA called microRNA (miRNA) is the focus of this paper. We will first briefly look at some of the characteristics and biogenesis of miRNA in plant systems. The remainder of the paper will go into details of three different approaches used to identify and study miRNA. These include two reverse genetics approaches: computation (bioinformatics) and experimental, and one rare forward genetics approach. We also will summarize how to measure and quantify miRNAs, and how to detect their possible targets in plants. Strengths and weaknesses of each methodological approach are discussed.

## 1. Introduction

### 1.1. MicroRNA Description

With the advent of more advanced molecular techniques, a newly discovered phenomenon of gene expression involving RNA has been characterized. MicroRNAs (miRNAs) are small endogenous, noncoding regulatory RNA sequences. They have been found to play key roles in regulatory functions of gene expression for most eukaryotes [1–3]. These endogenous RNA sequences are the interest of intensive research in various model organisms, ranging from plants to mammals. In plants, miRNAs are involved in a number of biological mechanisms including plant growth, development, and defense response against abiotic stress. Evidence indicates that miRNAs regulate gene expression at posttranscriptional levels in various organisms [3–6]. Further research has shown that miRNA sequences in plants are deeply conserved [5] and have near perfect complementarities with their specific messenger RNA (mRNA) targets. As a result of this complementarity, a plant miRNA guides the cleavage, degradation, or translational inhibition of its target mRNA, thereby affecting gene expression.

**Figure 1:**
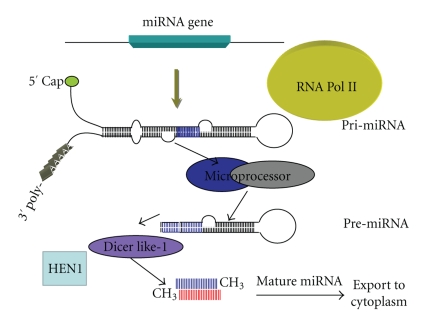
MicroRNA biogenesis.

Figure 1 and the following text illustrate the biogenesis of miRNAs. MicroRNAs are 21–24 nucleotides long and are processed in the nucleus from longer stem-loop structures called pre-miRNAs, that are approximately 70 nucleotides in length. miRNA genes are transcribed by RNA polymerase II, with some miRNA genes residing in intron sequences. After transcription, the 5′ end of pri-miRNA is capped and the 3′ end is polyadenylated. Based upon sequence homologies, the stem and loop structures are formed with base pairings. Pri-miRNA is thought to be longer than conserved stem-loop structures (pre-miRNA), therefore some processing takes place next.

Pri-miRNA is processed by an enzyme complex called *microprocessor* which includes Dicer like-1 nucleases (members of an RNase III endonuclease family) and HYL1, a dsRNA binding protein, bound to the pri-miRNA complex. In *Arabidopsis thaliana * all of the miRNA biogenesis steps are processed by one of the four Dicer-like RNase III endonucleases [7]. Among these, Dicer-like-1 is specialized for miRNA processing, while other Dicer-like enzymes are involved with another type of small RNA biogenesis and accumulation known as short interfering RNAs (siRNAs) [8]. However, Dicer like-4 dependence for the accumulation of several plant miRNAs has also been detected [9]. MicroRNAs are structurally and functionally similar to siRNA, but miRNAs originate from long stem loop double stranded RNAs (dsRNAs) [10].

At this point, the structure is now referred to as “pre-miRNA” and has one final step remaining. The pre-miRNA complex is methylated with HEN1 enzyme and then exported into the cytoplasm with the help of HASTY, an miRNA transporter. After methylation, the structure is called “mature miRNA”. The HASTY protein might be a plant ortholog of an animal enzyme known as Exportin-5, which has been shown in animal systems to also transport mature miRNAs to the cytoplasm [2, 11].

**Figure 2:**
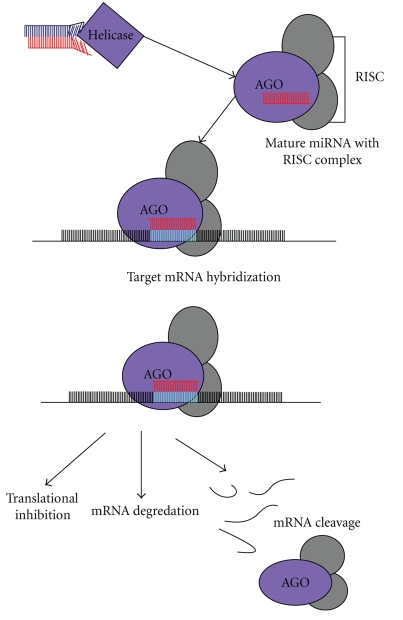
MicroRNA mechanism in plants.

The mature miRNA structure (miRNA) is next loaded into an RNA-induced ribonucleoprotein silencing complex (RISC) to cleave its specific target mRNA or to inhibit the translation of its target transcript (Figure 2) [12]. The RISC complex includes Arganoute (AGO1) (PAZ, RNA-binding domain and RNaseH-like P-element induced wimpy testis (PIWI) domain containing protein) and miRNA* is degraded. The AGO protein family is the most important and key component of the miRNA-RISC complex [7]. Their ability to suppress protein synthesis and association with miRNAs was demonstrated in human [13]. Single-stranded miRNA in the RISC is able to target a specific mRNA sequence, having sequence complementarity and by the Piwi domain. The AGO component can then cleave the miRNA-mRNA duplex (or siRNA-mRNA duplex) [7], thereby slowing gene expression of that particular mRNA.

On the other hand, translational inhibition and mRNA degradation are also other ways gene expression is regulated by miRNA. This can occur via deadenylation of the 3′ poly (A) tail and decapping of the 5′ end in mRNAs, which leads to progressive mRNA decay and degradation [14]. It has been demonstrated that in *Drosophyla melanogaster* S2 cells, the P-body protein GW182, which is a key component marking mRNAs for decay, interacts with the AGO1 [15]. Furthermore, RNA directed-DNA methylation revealing epigenetic regulation of gene expression has been demonstrated in *Arabidopsis*, reviewed in [16]. This process is initiated with RNA signal through cleavage of dsRNA by Dicer Like family 3 (DCL3) proteins. These signals target DNA methyltransferases to catalyse methylation of DNA [17]. Histone H3 lysine 9 is an important target for epigenetic modifications in plants. H3K9 methylation is associated with epigenetic regulation of gene expression and heterochromatin modification [18].

To summarize the basic principles of miRNA biogenesis, although we see many similarities between plant and animal systems, there are also ample differences between plant and animal miRNA characteristics and biogenesis.

First, it is known that plant miRNAs are mostly generated from noncoding transcriptional units [5] in contrast with some of the animal miRNAs which are processed from introns and protein coding genetic sequences [19]. Compared to animals, plants have a more complex small RNA population in their transcriptomes. Due to the abundance of plant-specific RNA Polymerase IV and RNA Polymerase V-dependent siRNA and *trans*-acting siRNAs, plant miRNAs are represented in the pool of small RNAs. By contrast, animal small RNA populations are generally filled with miRNAs in their transcriptomes [8]. Plant miRNAs have a unique 5′ end which differs from animal miRNA 5′ end sequences. To repress translation, plant miRNAs tend to bind to the protein-coding region of target mRNAs [20–22], but animal miRNAs bind to the 3′ untranslated region (3′ UTR) of their target mRNA transcripts [23].

To date, 1763 miRNAs have been identified in plants, including 187 from *Arabidopsis*, 377 from rice, 234 from Populus, 98 from maize, 72 from sorghum, 230 from Physcomitrella, 38 from *Medicago truncatula*, 78 from soybean, 37 from *Pinus taeda*, 58 from * Selaginella moellendorffii*, 44 from *Brassica napus* and 16 from sugar-cane (miRBase release 13.0, March, 2009, http://microrna.sanger.ac.uk/sequences/). These plant miRNAs have been identified via computational (bioinformatics) and/or experimental methods. For instance, through sequence homology analysis, 30 potential miRNAs were predicted from cotton [24] and an additional 58 wheat miRNAs have been identified by Yao et al. [25]. The majority of plant miRNAs studied to date negatively regulate their target gene expression at the posttranscriptional level. They are involved in regulating developmental processes [3, 26, 27] responding to environmental stresses [27, 28] and play a variety of important biological and metabolic processes [29–31]. Some examples of these processes include the regulation of plant development (miR172, floral organ specification, and miR166, leaf polarity), root initiation and development (ath-miR164), signal transduction (i.e., miR159, miR160, miR164, and miR167), and also plant environmental response (miR391 and miR395), as reviewed by Zhang et al. [32].

### 1.2. Strategies for miRNA Identification and Characterization

In reverse genetics strategies, researchers are utilizing known sequences to discover functions or phenotypes. miRNA identification largely relies on two main reverse genetics strategies: (1) computer-based (bioinformatics) and (2) experimental approaches. A third identification approach, forward genetics, is rarely used in miRNA discovery. Forward genetics is the classical approach where researchers have a known phenotype, but the DNA sequence (genotype) coding for that particular phenotype is unknown.

miRNA identification using bioinformatics tools is one of the most widely used methods, contributing considerably to the prediction of new miRNAs in both animal and plant systems. This is largely due to the low cost, high efficiency, fast and comprehensive methodology of bioinformatics. The main theory behind this approach is finding homologous sequences of known miRNAs both within a single genome and across genomes of related organisms [33, 34]. Sequence and structure homologies are used for computer-based predictions of miRNAs. Computational strategies provide a valuable and efficient manner to predict miRNA genes and their targets. The software-based approach is applied to animals, human, fungi, and plants [35–38]. For example Zhang et al. identified 338 new possible miRNAs in 60 different plant species [31] and Adai et al. have predicted 43 new miRNAs in *Arabidopsis* [39]. 

In contrast, cloning and sequencing of small RNA libraries represents an experimental approach to identify and characterize miRNAs. However, in contrast with bioinformatics, such approaches for miRNA identification also have limitations. First, most of the miRNAs are tissue and time specific, and generally their expression level is low. In addition, they mostly express in response to specific environmental stimuli. They also coexist with their cleaved and degraded target mRNAs, hence cloning small RNAs (miRNA and siRNA) is difficult, whereas computational approaches are effective because of no need for cloning. Since forward genetics, or the genetic screening approach, is time consuming, expensive, and less efficient, it is rarely used for plant miRNA identification. Next generation massive sequencing techniques such as pyrosequencing and Illumina are also applied to identify new miRNAs in plants [40, 41]. 

Here we summarize the main approaches to each strategy for identifying plant miRNAs, starting with computational (bioinformatics) approaches.

## 2. Computational Approach

### 2.1. Sequence and Structure Conservation in miRNAs

Once potential miRNA sequences have been cloned and sequenced, the sequence data can be imported into a variety of software programs for computational analysis. These bioinformatics tools search for sequence and structure conservation of miRNAs [42] using homology searches with previously known/identified miRNAs. To date a number of computational methods have been reported for the identification of plant miRNAs [5, 27, 33, 39, 43, 44]. Research in plants has revealed that short length sequences of mature miRNAs are conserved and have high complementarities to their target mRNAs [27]. Hence, candidate miRNAs can be detected using the conserved complementarities of miRNA to target mRNA, if the mRNA target sequence is known. On the other hand, it has also been shown that the secondary structures of miRNA precursor (pre-miRNA) are relatively more conserved than pri-miRNA sequences (precursor of pre-miRNA) (Figure 1) [45]. Recent bioinformatics tools were used to identify miRNA utilizing both sequence and secondary structure alignments; one of these tools is miRAlign in which more properties of miRNA structure conservation are considered (http://bioinfo.au.tsinghua.edu.cn/miralign) [43, 45]. Since the characteristic patterns of the conservation of miRNAs are searched by algorithms, the major challenge is finding miRNAs which are species specific and unrelated to previously known organisms.

### 2.2. Bioinformatics Tools Used for Identifying miRNA and Its Target mRNA

Several programs have been designed for the identification of miRNAs and their targets. Here we summarize five of the most commonly and widely used software tools for identifying miRNAs and miRNA targets. In this section, the softwares and databases are exemplified and their use in identifying plant miRNAs is described. The first one, miRBase, is currently a database of all known miRNA sequences. Following the description of miRBase, the plant miRNA-mRNA target finder called miRU will be explained. The secondary structure for a given pre-miRNA sequence can be predicted with appropriate criteria using a third software, RNAmFold. Another program, micro-HARVESTER, can be applied to find homology of a given miRNA in one plant species with a candidate miRNA in another plant species. Also mentioned in this section is findmiRNA, which is used for finding possible miRNAs in a given precursor miRNA sequence. After each of these software tools (described below), an example will be given.

#### 2.2.1. miRBase (http://microrna.sanger.ac.uk/)

miRBase is a central online database for all (plant, animal, virus, fungus to date) miRNAs including sequences, nomenclature, and target mRNA prediction data from all species. Currently the 13.0 version of the online database (March, 2009) consists of 8619 miRNA total entries from 103 species. These entries represent 9539 hairpin precursor miRNAs, expressing 9169 mature miRNA products with 1763 plant miRNAs. The database has three main functions. *miRBase::Registry* is where individual data is uploaded to the database prior to publication of novel miRNAs. *miRBase::Sequences* provides miRNA sequences, nomenclature, and references. *miRBase::Targets* provides the prediction of the mRNA target from all published animal miRNAs [46].

#### 2.2.2. miRU (http://bioinfo3.noble.org/miRNA/miRU.htm)

miRU is known as a potential plant mRNA target finder. Using this database, a mature miRNA sequence from a plant species is uploaded. The miRU system searches for potential complementary target sites in miRNA-target recognition with acceptable mismatches. Specifically, the user enters a mature miRNA sequence in the 5′ to 3′ direction (entered sequence can be in a range of 19–28 nucleotides long) then the dataset should be selected for prediction of mRNA target in the intended organism of interest. The allowable complementary mismatches between the target mRNA and the uploaded miRNA sequence can be adjusted or limited by the user. The output report provides information for each predicted miRNA target including gene identifier, target site position, mismatch score, number of mismatches, and target complementary sequence with color highlighted mismatches [31]. miRU is a very useful software for identifying mRNA targets of specific plant miRNAs. However not all plant species are available at this time.

#### 2.2.3. RNA mFold (http://rna.tbi.univie.ac.at/cgi-bin/RNAfold.cgi)

The algorithm utilized in the RNAmFold bioinformatics tool predicts secondary structures of single stranded RNA or DNA sequences. It is currently packaged in the Vienna RNA websuite, a collection of tools for folding, designing, and analyzing of RNA sequences [47]. The package also provides additional analysis of folding parts using the barriers program and structural RNA alignments. The package includes basic programs such as *RNAFold* for structure prediction of single sequences, *RNAalifold* for consensus miRNA structure prediction on a set of aligned sequences, *RNAinverse* for seqence design, *RNAcofold * and * RNAup* for RNA-RNA interaction analysis, *LocARNA* for the generation of structural alignment and barriers, and *treekin* for folding kinetics analysis. The RNAmFold tool is used for predicting the minimum free energy (MFE) secondary structure using the algorithm originally proposed by Zuker and Stiegler [48]. Equilibrium base-pairing probabilities of MFE structures are calculated via McCaskill's partition function (PF) algorithm [49].

#### 2.2.4. micro-HARVESTER (http://www-ab.informatik.unituebingen.de/brisbane/tb/index.php)

micro-HARVESTER is a computational tool that searches for miRNA homologs in a given miRNA sequence query. Due to sequence similarity, the search step is followed by a set of structural filters. This method is a sensitive approach to identify miRNA candidates with higher specificity. The approach uses a BLAST search to generate the first set of candidates and then the process continues with a series of filters based on structural features specific to plant miRNAs to achieve the desired specificity [50].

#### 2.2.5. findmiRNA (http://sundarlab.ucdavis.edu/mirna/) (A Resource of Predicted miRNA and Precursor Candidates for the Arabidopsis Genome)

findmiRNA algorithm is used for predicting potential miRNAs in a given set of candidate precursor sequences which have corresponding target sites in the transcriptome. Generally the algorithm is based on the complementarity existing between plant miRNAs and their mRNA targets to identify initial putative miRNA. Then the software analyzes the candidate miRNA precursor sequence with regard to forming a stem-loop structure [39]. Since the tool identifies any sequence with the potential to form hairpin structures, it has limitations such as the possibility of identifing tRNAs, foldback elements, and retrotransposons [39].

#### 2.2.6. MiRCheck (http://web.wi.mit.edu/bartel/pub/software.html)

MiRCheck is an algorithm designed to identify 20 mers which encode potential plant miRNAs [27]. Entries should be (1) putative miRNA hairpin sequences, (2) putative hairpin secondary structures, and (3) 20-mer potential plant miRNA sequences within the hairpin for MIRcheck algorithm. This software requires data of miRNA complementarities that should be conserved between homologous mRNAs in *Arabidopsis* and *Oryza sativa*. Researchers use this software to check their candidate miRNAs if they have potential to encode miRNA.

To briefly summarize, the largest limitation of most bioinformatic methods is the need to start from a known homologue and depend heavily on conservation of secondary structure and mature miRNA sequences. More advanced methods using hidden Markov models can overcome this limitation. As an example, Kadri et al. [51] have developed a novel approach, Hierarchical Hidden Markov Model (HHMM) that utilizes region-based structural information of miRNA precursors. They used this model for computational miRNA hairpin prediction in the absence of conservation in human [51].

### 2.3. EST Database Analysis Used for miRNA Prediction

Now we will describe how the above tools are utilized in the search of miRNAs. One such example is with Expressed sequence tags (ESTs). ESTs are partial sequences of complementary DNA (cDNA) cloned into plasmid vectors [52]. RNA is the starting material from which the cDNA clone is made, using *reverse transcriptase*. Many important plant genes using EST databases have been cloned [53, 54]. It is well known that miRNAs are deeply conserved from species to species, which allows researchers the ability to predict orthologues of previously known miRNAs by utilizing EST databases. Availability of ESTs in databases for identifying new plant miRNAs increases with coverage of the genome and number of sequences. Currently, GeneBank release 171.0 April 2009 (http://www.ncbi.nlm.nih.gov/Genbank/) contains 103 335 431 EST sequences, representing more than 1370 different organisms. The number of ESTs available for a specific organism can be found at http://www.ncbi.nlm.nih.gov/dbEST/dbEST_summary.html. This particular website is the best to utilize because conserved candidate miRNAs and their precursors can be predicted using this resource. The largest number of plant ESTs is from maize (*Zea mays*) (2 018 530), thale cress (*Arabidopsis thaliana*) (1 527 298), soybean (*Glycine max*) (1 386 618), rice (*Oryza sativa*) (1 248 955), wheat (*Triticum aestivum*) (1 064 111), oilseed rape (*Brassica napus*) (596 471), and barley (*Hordeum vulgare*) (525 527).

To identify homologous miRNAs across plant species, EST analysis approaches have been developed using sequence conservation of known miRNAs. An extra filtering which provides structure prediction (secondary structure), such as the “Zuker folding” algorithm with RNAmFold software [55], has also been applied [27, 31, 39].

Zhang et al. [31] reported an EST database analysis for predicting new plant miRNA genes using the BLAST algorithm to search known plant miRNAs (taken from miRBase 3.1 April, 2004). Their additional filter was the Zuker folding algorithm (mFold 3.1) to predict the secondary structure of putative miRNA sequences. The Zuker algorithm outputs used to analyze the results included the number of structures, free energy (ΔG kcal/mol), miRNA-like helicity, the number of arms per structure, size of helices within arms, and size and symmetry of internal loops within arms. Then hairpin stem-loop structures of predicted putative miRNAs were analyzed for structure filtering with known miRNAs using computational strategies [42]. The highest score of stem-loop structures was considered as new miRNAs and then the ESTs (with high similarity, *E* value less than e-100) were assigned as miRNA clones.

Jones-Rhoades and Bartel [10] have also analyzed EST databases to look for possible miRNAs in other plant species using known *Arabidopsis* miRNAs. They also revised the computational strategy with possible miRNA targets to increase sensitivity of the approach. Their first step for identifying miRNAs in the genome was detecting genomic portions containing imperfect inverted repeats using the “EINVERT” algorithm. Then they used the RNAmFold software to predict secondary structures of miRNA candidates. They checked all 20 mers within the inverted repeats against MiRCheck. After MiRCheck analysis they applied Patscan to identify 20 mers in *At*Set1 that matched at least one 20 mer in *Os*Set1 with 0–2 base substitutions. The Patscan algorithm was used in consideration of 20 mers on the same arm of their putative hairpins.

Bonnet et al. [35] also applied computational approaches to detect miRNAs and then applied EST analysis to confirm 91 newly identified miRNAs in *Oryza sativa* and *Arabidopsis thaliana*. Zhang et al. [31] have taken previously all-known *Arabidopsis* miRNAs (miRBase Release 3.0, April 2004) and searched the EST databases (using Basic Local Alignment Search Tool for nucleotide analyzes (BLASTn) 2.2.9 (May 1, 2004)) to find ESTs matched with miRNAs. They found a total of 18 694 BLAST hits in the databases and removed the EST sequences with high numbers (more than 2) of mismatched hits. Their result came to a total of 812 ESTs with 0 to 2 mismatches. They used those ESTs to predict secondary structures with RNA mFold software. Finally they identified 338 new potential miRNAs in 60 plant species.

### 2.4. Sample Method to Identify miRNA in a Plant Species Using Computational Approach

**Figure 3:**
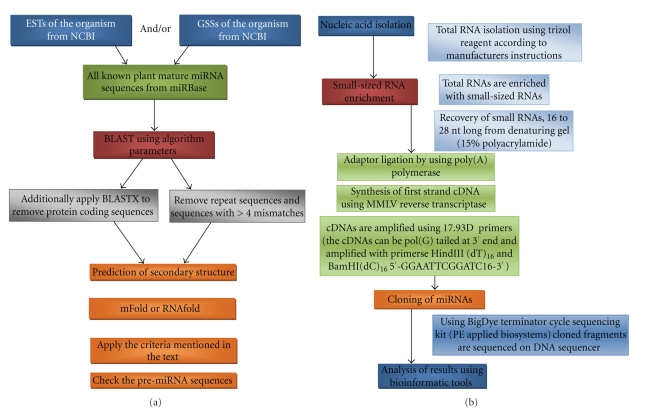
(a) Flow chart for computational approaches in identifying plant miRNAs. (b) Flow chart for experimental approaches in identifying plant miRNAs.

In this section, we work through an example of using ESTs and the software tools discussed previously, to identify plant miRNAs. In order to predict the plant miRNAs, EST sequences should be downloaded from the GenBank database (http://www.ncbi.nlm.nih.gov/) (Figure 3(a)). Searching for miRNA-like sequences includes two major procedures: searching pre-miRNA-like sequences and identifying pre-miRNAs and miRNAs. First, the RNAfold (http://rna.tbi.univie.ac.at/cgi-bin/RNAfold.cgi) [47] program or mFold program (http://frontend.bioinfo.rpi.edu/applications/mfold/cgi-bin/rna-form1.cgi) [55] can be used to find potential miRNA hairpin structures from the databased EST sequences [56]. According to Weibo et al. [57], strict criteria should be adopted in the identification of pre-miRNA-like sequences from hairpin structure sequences. These are

- 60 nucleotides is the minimum length of pre-miRNA sequences;
- the stem of the hairpin structure (including the GU wobble pairs) includes at least 17 base pairs;
−15 kcal/mol should be the maximum free energy of the secondary structure;
- the secondary structure must not compromise multibranch loops;
- the GC content of pre-miRNA should be between 24 and 71%.

Following these criteria assures that the processed sequences are similar to real pre-miRNAs, according to widely accepted characteristics. Second, the real pre-miRNAs might be identified from a large number of pre-miRNA-like sequences using a program such as GenomicSVM (http://geneweb.go3.icpcn.com/genomicSVM/). This program was developed using the “Support Vector Machine” model [57]. Alternatively, miRCheck (http://web.wi.mit.edu/bartel/pub/softwareWebTools.html) can be applied (Figure 3(a)) for confirming sequences to identify, if the entries contain 20 mers which encode potential plant miRNAs.

## 3. Experimental Approaches

Computational methods for identifying miRNAs in plants are rapid, less expensive and relatively easy compared with experimental procedures. However, these bioinformatic approaches can only identify conserved miRNAs among organisms and DNA or RNA sequence information is required in order to run the softwares. On the other hand, the computationally predicted miRNAs should also be confirmed via experimental methods. These experimental method options are described next.

### 3.1. Direct Cloning and Sequencing of Small RNA Libraries

Direct cloning of small RNAs from plants is one of the basic approaches of miRNA discovery. Scientists have used this methodology to isolate and clone small RNAs from various plant species such as *Arabidopsis* and rice [5, 20, 21, 58, 59]. Identification of miRNAs using the direct cloning approach basically involves the creation of a cDNA library and includes six steps: (1) isolation of total RNA from plant tissue, (2) recovery of small RNAs from an acrylamide gel, (3) adaptor ligation, (4) reverse transcription, RT-PCR, (5) cloning, and (6) sequencing methods. Expression of several miRNAs is broad but many of them are detected in certain environmental conditions, at different plant developmental stages and tissues. Therefore specific time points, tissues, and/or biotic and abiotic stressed induced plant samples are used for miRNA cloning. The most common plant species used for direct cloning are *Arabidopsis thaliana* [5, 58, 59], *Oryza sativa* (rice) [60], (cottonwood) [61], and *Triticum aestivum* (wheat) [24].

### 3.2. An Example of the Direct Cloning Experimental Approach

Total RNA is extracted from the organism of interest [40, 41]. Next, small RNAs approximately 16–28 nucleotides long are selected from the total RNA and excised from a polyacrylamide gel. Next, these small RNAs are ligated with an adaptor and reverse transcribed [62]. Resulting cDNAs are amplified with Real-Time PCR (RT-PCR) using primers designed for adaptor sites. Finally, the RT-PCR products are concatamerized and cloned [63]. Selected clones are sequenced and the sequence data is then analyzed (Figure 3(b)). These experimental procedures for identifying miRNA have been successfully applied and detailed by Elbashir et al. [63], Lau et al. [62], and Park et al. [58]. Alternatively, new high-throughput technologies such as 454 pyrosequencing and Solexa sequencing can be used for identification of plant miRNAs [40, 41].

The most important advantage of high-throughput deep sequencing technology compared to computational approaches is the opportunity for finding nonconserved and species specific miRNAs. To identify conserved and nonconserved miRNAs in tomato, Moxon et al. (2008) used the pyrosequencing approach [40]. On the other hand, Szittya et al. (2008) successfully used Solexa sequencing to find new miRNAs in barrel medic (*Medicago truncatula*) [41]. They have identified 25 conserved and 26 novel nonconserved miRNAs using 1 563 959 distinct sequences and 2 168 937 reads [41]. Experimental approaches also provide to detect and measure the specific miRNAs expressed in plants. Using the following methods plant miRNAs are efficiently detected and quantified.

### 3.3. miRNA Detection and Quantification Methods

Efficient and suitable miRNA detection and quantification are essential to understand miRNA function in specific conditions, cell and tissue types. Northern hybridization, cloning, and microarray analysis are widely used to detect and quantify miRNAs in plants, but these techniques are less sensitive and are not high throughput compared with quantitative real-time reverse transcription PCR (qRT-PCR) and end-poind PCR. Effective and sensitive qRT-PCR detection can circumvent these limitations. Several methods have been developed to detect and quantify miRNA for mammalian cells [64–66]. Recently Varkonyi-Gasic et al. [67] described a protocol for an end-point and real-time looped RT-PCR procedure. Their approach includes two steps. In the first step, a stem-loop RT primer is designed, following the strategy developed by Chen et al. [68] and is hybridized with the candidate miRNA. The second step includes the specific amplification of the miRNA, using a forward primer specific for the miRNA and a universal reverse primer, which is designed for the stem-loop RT primer sequence. The clues for designing the reverse RT primers and miRNA specific forward primers are that the specificity of stem-loop RT primers for a certain miRNA is conferred by a six nucleotide extension at the 3′ end. This extension is the reverse complement of the last six nucleotides at the 3′ end of the miRNA. Forward RT primers are specifically designed for individual miRNA sequences. At the primer's 5′ end 5–7 random and relatively GC-rich nucleotides are added to increase the template's melting temperature [67].

To perform end-point and real-time looped RT-PCR miRNA quantification experiments, total RNA is isolated from a plant sample using the TRizol reagent according to manufacturer's protocol (Invitrogen, Carlsbad, CA). Then the stem-loop RT PCR reaction is performed by mixing the component as follows: 0.5 *μ*L 10 mM dNTP mix, 11.15 *μ*L nuclease-free water, 1 *μ*L of appropriate stem-loop RT primer (1 *μ*M), and the mix is heated at 65°C for 5 minutes and chilled on ice for 2 minutes. Additional components are added to the mixture, 4 *μ*L 5 × First-Strand buffer, 2 *μ*L 0.1 M DTT, 0.1 *μ*L RNaseOUT (40 units/*μ*L), and 0.25 *μ*L, SuperScript III RT (200 units/*μ*L). Finally the pulsed RT reaction incubation is set up as 30 minutes at 16°C, followed by pulsed RT of 60 cycles at 30°C for 30 seconds, 42°C for 30 seconds, and 50°C for 1 second. RT products can be used to detect and quantify individual miRNAs in plants via three different strategies, end-point PCR, SYBR Green I assay, and TaqMan UPL procedure. Each of these strategies is described next.

#### 3.3.1. End-Point PCR

A nontemplate control should be included with each experiment to insure the expected banding pattern for specific cDNA of miRNA amplification. A PCR master mix is prepared and the following components added to nuclease-free eppendorf tubes: 15.4 *μ*L nuclease-free water, 2 *μ*L 10 × PCR buffer, 0.4 *μ*L 10 mM dNTP mix, 0.4 *μ*L forward primer (10 *μ*M), 0.4 *μ*L reverse primer (10 *μ*M), and 0.4 *μ*L Advantage 2 Polymerase mix. Then 19 *μ*L of the PCR master mix should be aliquot into different tubes and 1 *μ*L RT product is added to reaction mixtures. After that, the thermal cycler is set up as 94°C for 2 minutes, followed by 20–40 cycles of 94°C for 15 seconds and 60°C for 1 minute. Finally the PCR reaction products are analyzed by electrophoresis on a 4% agarose gel in 1 × TAE gel.

#### 3.3.2. SYBR Green I Assay

SYBR Green I master mix is prepared according to real-time qPCR system (5 × LightCycler for Roche Diagnostics or 2X Master mix for Stratagene Mx3005p) by adding 1 *μ*L forward primer (10 *μ*M), 1 *μ*L reverse primer (10 *μ*M), 12 *μ*L nuclease-free water (for 5 × LightCycler for Roche Diagnostics) or (6 *μ*L nuclease-free water for 2X Master mix for Stratagene Mx3005p), 4 *μ*L SYBR Green I master mix for 5 × LightCycler for Roche Diagnostics or (10 *μ*L SYBR Green I master mix for Stratagene Mx3005p). Using nuclease-free eppendorf tubes for each qPCR reactions, 18 *μ*L prepared mixtures containing master mix and primers are pipetted into each tube. Adding 2 *μ*L RT products to tubes, the reaction is started. qPCR machine is set up as 95°C for 5 minutes, followed by 35–45 cycles of 95°C for 5 seconds, 60°C for 10 seconds, and 72°C for 1 second. For melting curve analysis samples are denaturated at 95°C, and then cooled to 65°C at 20°C per second. Fluorescence signals are collected at 530 nm wavelengths continuously from 65°C to 95°C at 0.2°C per second. Finally results are analyzed using the LightCycler or Stratagene software.

#### 3.3.3. miRNA TaqMan UPL Probe Procedure

To perform a TaqMan assay for miRNA detection and quantification using Universal Probe Library (UPL) probes, first a 5 × LightCycler TaqMan master mix (Roche Diagnostics) is prepared according to manufacturer's instructions. Next the following components are added to a nuclease-free eppendorf tube: 11.8 *μ*L nuclease-free water, 4 *μ*L TaqMan master mix, 1 *μ*L forward primer (10 *μ*M), 1 *μ*L reverse primer (10 *μ*M), 0.2 *μ*L UPL probe no. 21 (10 *μ*M). Then real-time qRT-PCR is performed with cycling temperatures and resulting data analyzed as described above with the SYBR Green I assay protocol. An educational eLesson and animation further describing the real time PCR technique can be found at the Plant and Soil Sciences eLibrary (http://plantandsoil.unl.edu/croptechnology2005/pages/index.jsp?what=topicsD&topicOrder=1&informationModuleId=1057077340).

### 3.4. Forward Genetics

miRNAs were first discovered via mutant analysis [64] in animals. However to date, there is only one example using a forward genetics experimental approach to identify miRNA in plants. Baker et al. [69] identified an miRNA loss of function allele by a transposon insertion upstream of the predicted *MIR164c* stem-loop. The miRNA mutant resulted in a flower phenotype with extra petals. Since highly conserved plant miRNAs are encoded by gene families, functional redundancy restricts the loss of function of an miRNA gene, making mutation searches highly inefficient. Overexpression of miRNA genes and precursors or construction of miRNA resistant transgenic plants have the potential to better provide a clear assessment of overlapping functions of other miRNA family members. If this comes to fruitition, then forward genetics approaches may become more viable in identifying miRNAs.

## 4. Identification of miRNA Targets

So far we have described computational (bioinformatics) and experimental approaches used to identify miRNA sequences. Now we will describe methods utilized to identify their targets, mRNA sequences cleaved or targeted by miRNA. Specific miRNA targets in plant genomes and transcriptomes have been identified with both experimental and computational approaches. Predicting miRNA targets in plants is much easier due to the high and significant complementarities to miRNA-mRNA targets [34]. The ability for plant miRNA to target mRNA with perfect sequence complementary matches was first shown with miR171 [59]. It was shown that miR171 has perfect antisense complementarity with three SCARECROW-like (SCL) transcription factors in the *Arabidopsis* genome. Additionally, this particular miRNA is transcribed from an intergenic locus and lacks a stem-loop structure [5, 21]. Predicting conserved miRNA targets in different organisms has revealed that homologous mRNAs are targeted by conserved miRNAs within an miRNA family, yet allowing more gaps and more mismatches between an individual miRNA and its target [27]. Next we will summarize some of the bioinformatic and experimental methods utilized to find mRNA targets of known miRNAs in plants.

### 4.1. Computer-Based Procedures for Predicting mRNA Sequences Targeted by miRNA

Several algorithms are used for predicting putative miRNA-mRNA targets in plants; for this purpose mirU is one of the widely used softwares. The mirU system using given miRNA sequences searches for potential mRNA targets with tolerable mismatches [70]. Additionally, Jones-Rhoades and Bartel [10] have developed a more refined method by using the MIR check algorithm to predict miRNA targets specifically in *Arabidopsis* and *Oryza sativa*. The MirCheck software allows for more mismatches and gaps in miRNA-mRNA complexes in these two species. This software also needs miRNA complementarities that should be conserved between homologous mRNAs in *Arabidopsis* and *Oryza sativa* [10]. They have scored the miRNA complementary sites as 0.5 for G:U wobble pairs, 1 for non-G:U wobble pairs, 2 for each bulged or loop nucleotide in the miRNA or target site. They have reported scored complementary results of ≤ 2 in conserved miRNA target mRNA sites in both *Arabidopsis* and *O. sativa*.

### 4.2. Experimental Approaches for Prediction of miRNA Targets

As with computational approaches, experimental approaches have been utilized widely to predict plant miRNA-mRNA target sites. Genome-wide expression profiling to search for miRNA targets can be applied on expression arrays. In one example, array data showed that five transcripts encoding *TCP* genes were downregulated via overexpression of miR319a (miR-JAW) in *Arabidopsis*. Those five TCP transcription factor mRNAs show up to five mismatches, or four mismatches when G:U wobble counts 0.5 mismatch [71]. Additionally Schwab et al. [22] overexpressed four different miRNAs in each *Arabidposis * plant and examined each expression profile to experimentally establish parameters for target cleavage guided by plant miRNAs. However, they found no new target mRNAs other than previously identified by computational approaches. Two new targets, not found through bioinformatics, were detected, but their cleaved products were not confirmed via 5′ RACE experiments.

### 4.3. 5′ RACE Experiment

At present, the most powerful method to confirm miRNA-mRNA targets is the 5′ RACE procedure (Random Amplification of cDNA Ends). 5′ RACE has been used by many researchers to identify miRNA targets in plants [3, 21, 71, 72]. Cleaved mRNA products in plants have two diagnostic properties. One is that the 5′ phosphate of a cleaved mRNA product can be ligated to an RNA adaptor with T4 RNA ligase. Second, in general, the precise target cleavage position is that mRNA target nucleotides pair with the tenth nucleotide of miRNA [21, 73]. Cleaved mRNA products by miRNA guided activity can be amplified with ligation of an oligo-nucleotide adaptor to the 5′ end, followed by reverse transcription and PCR amplification with a gene specific primer [21]. A modified 5′ RACE procedure can be applied as follows. Total RNA is isolated and polyA mRNA is prepared (Qiatex mRNA midi kit, Qiagen, CA) and directly ligated to an RNA oligo adaptor (supplied by GeneRacer kit, Invitrogen, CA). Oligo dT is used to synthesize the first strand of cDNA with reverse transcriptase. This first cDNA strand is amplified with GenRacer 5′ and 3′ primers for nongene specific amplification (according to manufacturer's procedures, Invitrogen, CA, USA or Clontech, RL, USA). Then the 5′ RACE PCR and 5′ nested PCR are performed using specific primers supplied with kits. RACE products are gel purified, cloned, and sequenced.

## 5. Concluding Remarks

miRNA studies in plants have already explained a number of biological events in response to both biotic and abiotic stresses. Improved understanding of molecular mechanisms of miRNA in plants will lead to the development of novel and more precise techniques that will help better understanding some posttranscriptional gene silencing in response to both biotic and abiotic stresses. Accumulating knowledge on the roles of plant miRNA's in molecular biology is leading to the development of more efficient and reliable tools for their characterization. Building new algorithms and models will overcome drawbacks and limitations of current softwares used for miRNA and target mRNA identification. New bioinformatic tools should be generated to separate miRNA hairpin structures from tRNAs and retrotransposons and to differentiate miRNAs from siRNAs. New algorithms or new models to help cleaved mRNA product analysis for target identification also require improvement. Considering the discovery that miRNAs play important roles in numerous biological events, greater research in this area is being stimulated.

## References

[1] Bartel B, Bartel DP (2003). MicroRNAs: at the root of plant development?. *Plant Physiology*.

[2] Bartel DP (2004). MicroRNAs: genomics, biogenesis, mechanism, and function. *Cell*.

[3] Mallory AC, Reinhart BJ, Jones-Rhoades MW (2004). MicroRNA control of *PHABULOSA* in leaf development: importance of pairing to the microRNA 5′ region. *The EMBO Journal*.

[4] Grishok A, Pasquinelli AE, Conte D (2001). Genes and mechanisms related to RNA interference regulate expression of the small temporal RNAs that control *C. elegans* developmental timing. *Cell*.

[5] Reinhart BJ, Weinstein EG, Rhoades MW, Bartel B, Bartel DP (2002). MicroRNAs in plants. *Genes and Development*.

[6] Chapman EJ, Prokhnevsky AI, Gopinath K, Dolja VV, Carrington JC (2004). Viral RNA silencing suppressors inhibit the microRNA pathway at an intermediate step. *Genes and Development*.

[7] Filipowicz W, Bhattacharyya SN, Sonenberg N (2008). Mechanisms of post-transcriptional regulation by microRNAs: are the answers in sight?. *Nature Reviews Genetics*.

[8] Meyers BC, Axtell MJ, Bartel B (2008). Criteria for annotation of plant microRNAs. *Plant Cell*.

[9] Rajagopalan R, Vaucheret H, Trejo J, Bartel DP (2006). A diverse and evolutionarily fluid set of microRNAs in *Arabidopsis thaliana*. *Genes and Development*.

[10] Jones-Rhoades MW, Bartel DP (2004). Computational identification of plant MicroRNAs and their targets, including a stress-induced miRNA. *Molecular Cell*.

[11] Yi R, Qin Y, Macara IG, Cullen BR (2003). Exportin-5 mediates the nuclear export of pre-microRNAs and short hairpin RNAs. *Genes and Development*.

[12] Hammond SM, Bernstein E, Beach D, Hannon GJ (2000). An RNA-directed nuclease mediates post-transcriptional gene silencing in Drosophila cells. *Nature*.

[13] Pillai RS, Artus CG, Filipowicz W (2004). Tethering of human Ago proteins to mRNA mimics the miRNA-mediated repression of protein synthesis. *RNA*.

[14] Parker R, Song H (2004). The enzymes and control of eukaryotic mRNA turnover. *Nature Structural and Molecular Biology*.

[15] Behm-Ansmant I, Rehwinkel J, Doerks T, Stark A, Bork P, Izaurralde E (2006). mRNA degradation by miRNAs and GW182 requires both CCR4:NOT deadenylase and DCP1:DCP2 decapping complexes. *Genes and Development*.

[16] Matzke MA, Birchler JA (2005). RNAi-mediated pathways in the nucleus. *Nature Reviews Genetics*.

[17] Cao X, Aufsatz W, Zilberman D (2003). Role of the DRM and CMT3 methyltransferases in RNA-directed DNA methylation. *Current Biology*.

[18] Zhou D-X (2009). Regulatory mechanism of histone epigenetic modifications in plants. *Epigenetics*.

[19] Baskerville S, Bartel DP (2005). Microarray profiling of microRNAs reveals frequent coexpression with neighboring miRNAs and host genes. *RNA*.

[20] Mette MF, Aufsatz W, Kanno T (2005). Analysis of double-stranded RNA and small RNAs involved in RNA-mediated transcriptional gene silencing. *Methods in Molecular Biology*.

[21] Sunkar R, Girke T, Jain PK, Zhu J-K (2005). Cloning and characterization of microRNAs from rice. *Plant Cell*.

[22] Schwab R, Palatnik JF, Riester M, Schommer C, Schmid M, Weigel D (2005). Specific effects of microRNAs on the plant transcriptome. *Developmental Cell*.

[23] Seggerson K, Tang L, Moss EG (2002). Two genetic circuits repress the *Caenorhabditis elegans* heterochronic gene *lin-28* after translation initiation. *Developmental Biology*.

[24] Zhang B, Wang Q, Wang K (2007). Identification of cotton microRNAs and their targets. *Gene*.

[25] Yao Y, Guo G, Ni Z (2007). Cloning and characterization of microRNAs from wheat (*Triticum aestivum* L.). *Genome Biology*.

[26] Guo H-S, Xie Q, Fei J-F, Chua N-H (2005). MicroRNA directs mRNA cleavage of the transcription factor *NAC1* to downregulate auxin signals for *Arabidopsis* lateral root development. *Plant Cell*.

[27] Laufs P, Peaucelle A, Morin H, Traas J (2004). MicroRNA regulation of the CUC genes is required for boundary size control in *Arabidopsis* meristems. *Development*.

[28] Sunkar R, Zhu J-K (2004). Novel and stress regulated microRNAs and other small RNAs from *Arabidopsis*. *Plant Cell*.

[29] Carrington JC, Ambros V (2003). Role of microRNAs in plant and animal development. *Science*.

[30] Ambros V, Chen X (2007). The regulation of genes and genomes by small RNAs. *Development*.

[31] Zhang BH, Pan XP, Wang QL, Cobb GP, Anderson TA (2005). Identification and characterization of new plant microRNAs using EST analysis. *Cell Research*.

[32] Zhang B, Pan X, Cobb GP, Anderson TA (2006). Plant microRNA: a small regulatory molecule with big impact. *Developmental Biology*.

[33] Lagos-Quintana M, Rauhut R, Lendeckel W, Tuschl T (2001). Identification of novel genes coding for small expressed RNAs. *Science*.

[34] Lee RC, Ambros V (2001). An extensive class of small RNAs in *Caenorhabditis elegans*. *Science*.

[35] Bonnet E, Wuyts J, Rouzé P, van de Peer Y (2004). Detection of 91 potential conserved plant microRNAs in *Arabidopsis thaliania* and *Oryza sativa* identifies important target genes. *Proceedings of the National Academy of Sciences of the United States of America*.

[36] Rhoades MW, Reinhart BJ, Lim LP, Burge CB, Bartel B, Bartel DP (2002). Prediction of plant microRNA targets. *Cell*.

[37] Lim LP, Glasner ME, Yekta S, Burge CB, Bartel DP (2003). Vertebrate microRNA genes. *Science*.

[38] Lewis BP, Shih I-H, Jones-Rhoades MW, Bartel DP, Burge CB (2003). Prediction of mammalian MicroRNA targets. *Cell*.

[39] Adai A, Johnson C, Mlotshwa S (2005). Computational prediction of miRNAs in *Arabidopsis thaliana*. *Genome Research*.

[40] Moxon S, Jing R, Szittya G (2008). Deep sequencing of tomato short RNAs identifies microRNAs targeting genes involved in fruit ripening. *Genome Research*.

[41] Szittya G, Moxon S, Santos DM (2008). High-throughput sequencing of *Medicago truncatula* short RNAs identifies eight new miRNA families. *BMC Genomics*.

[42] Lai EC, Tomancak P, Williams RW, Rubin GM (2003). Computational identification of Drosophila microRNA genes. *Genome Biology*.

[43] Wang J-W, Wang L-J, Mao Y-B, Cai W-J, Xue H-W, Chen X-Y (2005). Control of root cap formation by MicroRNA-targeted auxin response factors in *Arabidopsis*. *Plant Cell*.

[44] Wang XJ, Reyes JL, Chua NH, Gaasterland T (2004). Prediction and identification of *Arabidopsis thaliana* microRNAs and their mRNA targets. *Genome Biology*.

[45] Wang X, Zhang J, Li F (2005). MicroRNA identification based on sequence and structure alignment. *Bioinformatics*.

[46] Griffiths-Jones S, Saini HK, van Dongen S, Enright AJ (2008). miRBase: tools for microRNA genomics. *Nucleic Acids Research*.

[47] Gruber AR, Lorenz R, Bernhart SH, Neuböck R, Hofacker IL (2008). The Vienna RNA websuite. *Nucleic Acids Research*.

[48] Zuker M, Stiegler P (1981). Optimal computer folding of large RNA sequences using thermodynamics and auxiliary information. *Nucleic Acids Research*.

[49] McCaskill JS (1990). The equilibrium partition function and base pair binding probabilities for RNA secondary structure. *Biopolymers*.

[50] Dezulian T, Remmert M, Palatnik JF, Weigel D, Huson DH (2006). Identification of plant microRNA homologs. *Bioinformatics*.

[51] Kadri S, Hinman V, Benos PV (2009). HHMMiR: efficient de novo prediction of microRNAs using hierarchical hidden Markov models. *BMC Bioinformatics*.

[52] Adams MD, Kelley JM, Gocayne JD (1991). Complementary DNA sequencing: expressed sequence tags and human genome project. *Science*.

[53] Graham MA, Silverstein KAT, Cannon SB, VandenBosch KA (2004). Computational identification and characterization of novel genes from legumes. *Plant Physiology*.

[54] Jung JD, Park H-W, Hahn Y (2003). Discovery of genes for ginsenoside biosynthesis by analysis of ginseng expressed sequence tags. *Plant Cell Reports*.

[55] Zuker M (2003). Mfold web server for nucleic acid folding and hybridization prediction. *Nucleic Acids Research*.

[56] Hofacker IL (2003). Vienna RNA secondary structure server. *Nucleic Acids Research*.

[57] Weibo J, Nannan L, Bin Z (2008). Identification and verification of microRNA in wheat (*Triticum aestivum*). *Journal of Plant Research*.

[58] Park W, Li J, Song R, Messing J, Chen X (2002). CARPEL FACTORY, a Dicer homolog, and HEN1, a novel protein, act in microRNA metabolism in *Arabidopsis thaliana*. *Current Biology*.

[59] Llave C, Xie Z, Kasschau KD, Carrington JC (2002). Cleavage of *Scarecrow-like* mRNA targets directed by a class of *Arabidopsis* miRNA. *Science*.

[60] Sunkar R, Girke T, Zhu J-K (2005). Identification and characterization of endogenous small interfering RNAs from rice. *Nucleic Acids Research*.

[61] Lu S, Sun Y-H, Shi R, Clark C, Li L, Chiang VL (2005). Novel and mechanical stress-responsive MicroRNAs in *Populus trichocarpa* that are absent from *Arabidopsis*. *Plant Cell*.

[62] Lau NC, Lim LP, Weinstein EG, Bartel DP (2001). An abundant class of tiny RNAs with probable regulatory roles in *Caenorhabditis elegans*. *Science*.

[63] Elbashir SM, Martinez J, Patkaniowska A, Lendeckel W, Tuschl T (2001). Functional anatomy of siRNAs for mediating efficient RNAi in *Drosophila melanogaster* embryo lysate. *The EMBO Journal*.

[64] Lee RC, Feinbaum RL, Ambros V (1993). The *C. elegans* heterochronic gene *lin-4* encodes small RNAs with antisense complementarity to *lin-14*. *Cell*.

[65] Tang F, Hajkova P, Barton SC, Lao K, Surani MA (2006). MicroRNA expression profiling of single whole embryonic stem cells. *Nucleic Acids Research*.

[66] Schmittgen TD, Lee EJ, Jiang J (2008). Real-time PCR quantification of precursor and mature microRNA. *Methods*.

[67] Varkonyi-Gasic E, Wu R, Wood M, Walton EF, Hellens RP (2007). Protocol: a highly sensitive RT-PCR method for detection and quantification of microRNAs. *Plant Methods*.

[68] Chen C, Ridzon DA, Broomer AJ (2005). Real-time quantification of microRNAs by stem-loop RT-PCR. *Nucleic Acids Research*.

[69] Baker CC, Sieber P, Wellmer F, Meyerowitz EM (2005). The *early extra petals1* mutant uncovers a role for microRNA miR164c in regulating petal number in *Arabidopsis*. *Current Biology*.

[70] Zhang Y (2005). miRU: an automated plant miRNA target prediction server. *Nucleic Acids Research*.

[71] Palatnik JF, Allen E, Wu X (2003). Control of leaf morphogenesis by microRNAs. *Nature*.

[72] Mallory AC, Bartel DP, Bartel B (2005). MicroRNA-directed regulation of *Arabidopsis AUXIN RESPONSE FACTOR17* is essential for proper development and modulates expression of early auxin response genes. *Plant Cell*.

[73] Kasschau KD, Xie Z, Allen E (2003). P1/HC-Pro, a viral suppressor of RNA silencing, interferes with *Arabidopsis* development and miRNA function. *Developmental Cell*.

